# Decreased mannan-binding lectin level in adults with hypopituitarism; dependence on appropriate hormone replacement therapies

**DOI:** 10.3389/fimmu.2023.1107334

**Published:** 2023-04-12

**Authors:** Aleksandra E. Matusiak, Jan Stępniak, Andrzej Lewiński, Małgorzata Karbownik-Lewińska

**Affiliations:** ^1^ Department of Endocrinology and Metabolic Diseases, Polish Mother’s Memorial Hospital – Research Institute, Lodz, Poland; ^2^ Department of Oncological Endocrinology, Medical University of Lodz, Lodz, Poland; ^3^ Department of Endocrinology and Metabolic Diseases, Medical University of Lodz, Lodz, Poland

**Keywords:** mannan-binding lectin (MBL), complement system, lectin pathway, hypopituitarism, endocrine disorders

## Abstract

**Background:**

Mannan-binding lectin (MBL) is a main component of the lectin pathway of the complement system. Although there are some studies showing links between endocrine and immune systems, the ones concerning hypopituitarism are limited. The aim of this study was to check whether there is any association between blood MBL level and pituitary hormone deficiencies and whether this relationship is affected by appropriate hormone replacement therapies.

**Methods:**

One hundred and twenty (120) inpatients, aged 18-92, were divided into two main groups, *i.e.* control individuals (21/120) and patients with pituitary diseases (99/120). The latter were diagnosed either with hypopituitarism (n=42) or with other pituitary diseases (not causing hypopituitarism) (n=57). Additionally, hypopituitary patients on appropriate replacement therapies (compensated hypopituitarism) were compared to patients on inappropriate replacement therapies (non-compensated hypopituitarism). Several parameters in blood serum were measured, including MBL level, pituitary and peripheral hormones and different biochemical parameters.

**Results:**

Serum MBL level was significantly lower in patients with hypopituitarism comparing to controls (1358.97 ± 244.68 vs. 3199.30 ± 508.46, *p*<0.001) and comparing to other pituitary diseases (1358.97 ± 244.68 vs. 2388.12 ± 294.99, *p=*0.015) and this association was confirmed by univariate regression analysis. We evaluated the distribution of patients with relation to MBL level; there was a clear difference in this distribution between control individuals (among whom no subjects had MBL level <500 ng/mL) and patients with hypopituitarism (among whom 43% of patients had MBL level <500 ng/mL). Moreover, patients with non-compensated hypopituitarism had lower mean and median MBL levels comparing to patients with compensated hypopituitarism (1055.38 ± 245.73 vs. 2300.09 ± 579.93, *p=*0.027; 488.51 vs. 1951.89, *p=*0.009, respectively) and this association was confirmed in univariate regression analysis. However, mean and median MBL levels in patients with compensated hypopituitarism vs. controls did not differ significantly (2300.09 ± 579.93 vs. 3199.30 ± 508.46, *p=*0.294; 1951.90 vs. 2329.16; *p=*0.301, respectively).

**Conclusion:**

Hypopituitarism in adults is associated with a decreased blood concentration of mannan-binding lectin, a phenomenon which does not exist in hypopituitary patients on the appropriate hormone replacement therapies. Therefore measurement of mannan-binding lectin level in patients with hypopituitarism may be considered as a parameter contributing to adjust optimal doses of hormone replacement therapies.

## 1 Introduction

The complement system is an important effector arm of innate immunity and plays a crucial role in the defense against common pathogens. Effective defense and maintenance of homeostasis requires a perfect balance between complement activation and control.

Mannan-binding lectin (MBL, also referred to as mannose-binding lectin), which constitutes a main component of the lectin pathway of the complement system, is an immune defense plasma protein synthesized in the liver and its basal concentration in human plasma is primarily genetically determined (1, 2). MBL plays a crucial role in the immune response (2).

Although the concentration of MBL is genetically determined, there are some other factors affecting MBL level such as bacterial infection, for example caused by *Streptococcus pneumoniae* (3) or viral infection resulting in hepatitis C (4) or COVID-19 (5).

There are studies showing the existence of casual links between the endocrine and the immune system. MBL is one possible connection between these fields. Of great importance are findings showing association of MBL level with thyroid dysfunction. Increased MBL blood concentrations were found in hyperthyroid, while decreased MBL levels were found in hypothyroid (6, 7) patients. Even high-normal TSH (>2.5 mIU/L) in women of childbearing age, which is commonly considered to be just abnormal in this specific population, was associated with lower MBL concentrations (8). Clinical findings also indicate that growth hormone (GH) influences the concentration of MBL in blood plasma (9, 10). Baseline concentrations of MBL were found to be lower in GH deficient patients and higher in patients with acromegaly (9). Moreover, treatment with rhGH significantly increased MBL concentrations in healthy and GH-deficient subjects (9). Expectedly, the treatment with pegvisomant (GH receptor antagonist) or with octreotide (somatostatin analog) in acromegalic patients decreased MBL level (9). Also stimulatory effects of growth hormone and thyroid hormones on MBL synthesis in the human hepatocyte cell line were documented (11). According to our knowledge there are no published data regarding effects of other pituitary tropic hormones (or their deficiencies) on complement lectin pathway. Therefore published evidence on connections between endocrine system and lectin pathway is rather poor and our study does constitute a next step to broaden this field.

The aim of the present study was to investigate whether pituitary hormone deficiencies (hypopituitarism) influence MBL blood level and whether MBL concentration may be changed in response to appropriate hormone replacement therapies in patients with hypopituitarism. Also a possible relationship between MBL and other laboratory parameters, which may be affected by pituitary dysfunction, was examined.

## 2 Materials and methods

The Ethical Committee of the Polish Mother’s Memorial Hospital - Research Institute, Lodz, Poland approved all the procedures applied in the present study, and fully informed written consent was obtained from all patients (No. 47/2019, 87/2022).

One hundred and twenty (120) inpatients (89 females, 74%), aged 18-92, who were admitted to the hospital because any pituitary disease was suspected, were enrolled to the study in the years 2019-2022. All participants were patients of the Department of Endocrinology and Metabolic Diseases, Polish Mother’s Memorial Hospital - Research Institute, Lodz, Poland. Individuals were not randomized into groups and blinding was not performed because our study is not a randomized controlled trial (and individuals simply could not ethically be randomized and blinded).

The patients were divided into two main groups:
− Controls – twenty one out of 120 subjects (21/120; 17.5%) with non-secreting pituitary microadenomas, Empty Sella Syndrome and pituitary cysts [the two latter not causing pituitary dysfunction] and with no other endocrine diseases; therefore no therapeutical intervention was required and these patients were treated as healthy individuals;
− Patients with pituitary diseases – ninety nine out of 120 subjects (99/120; 82.5%). All these patients required any endocrine treatment.
The second group of 99 patients was further divided into two following groups:
- forty two (42) out of 120 subjects (42/120; 35%) diagnosed with hypopituitarism of different degree, *i.e.* with different number of pituitary deficiencies;
- fifty seven (57) out of 120 subjects (57/120; 47.5%) with other pituitary diseases (not causing hypopituitarism of any degree) such as secreting microadenomas or non-secreting and secreting macroadenomas; patients after pituitary surgery or after radiotherapy (but without pituitary insufficiency); two patients with *PROP-1* gene mutation; one patient with Schaaf-Yang syndrome; also patients with non-secreting microadenomas or Empty Sella Syndrome and with coexisting primary hypothyroidism.
Patients with hypopituitarism (n=42) were divided into two following groups:
- patients who were on appropriate hormone replacement therapies (compensated hypopituitarism; n=10)
- patients who were not on appropriate hormone replacement therapies (non-compensated hypopituitarism; n=32).

We evaluated the accuracy of hormone replacement therapies by checking if levels of the individual end-organ hormones are in target ranges, according to the current guidelines (12).

Patients with hypopituitarism (n=42) had the following pituitary deficiencies:
1. one pituitary deficiency (n=9); compensated (n=5) vs. non-compensated (n=4):
- isolated GH deficiency (Shaaf-Yang Syndrome), 1 patient; non-compensated
- isolated LH/FSH deficiency (hypogonadotropic hypogonadism due to macroprolactinoma), 1 patient; non-compensated
- isolated TSH deficiency (one patient with pituitary apoplexy being secondary to abrupt hemorrhage or infarction of pituitary adenoma and two idiopathic cases confirmed in thyrotropin-releasing hormone (TRH) stimulation test), 3 patients; compensated
- isolated ACTH deficiency (two cases after surgery due to Cushing disease and two cases of the empty sella syndrome), 4 patients; compensated (n=2) vs. non-compensated (n=2)
2. two pituitary deficiencies (n=7); compensated (n=1) vs. non-compensated (n=6):
- LH/FSH and GH deficiency (2 patients); non-compensated
- LH/FSH and TSH deficiency (3 patients); compensated (n=1) vs. non-compensated (n=2)
- TSH and ACTH deficiency (2 patients); non-compensated
3. three pituitary deficiencies (n=9); compensated (n=2) vs. non-compensated (n=7):
- GH, LH/FSH and TSH (3 patients); non-compensated
- GH, LH/FSH and ACTH deficiency (3 patients); compensated (n=1) vs. non-compensated (n=2)
- GH, TSH and ACTH deficiency (1 patient); non-compensated
- LH/FSH, TSH and ACTH deficiency (2 patients); compensated (n=1) vs. non-compensated (n=1);
4. four pituitary (GH, LH/FSH, TSH, ACTH) deficiencies (n=12); compensated (n=2) vs. non-compensated (n=10)
5. five pituitary (GH, LH/FSH, TSH, ACTH, Prl) deficiencies (panhypopituitarism) (n=5); non-compensated.

**Figure 1:**
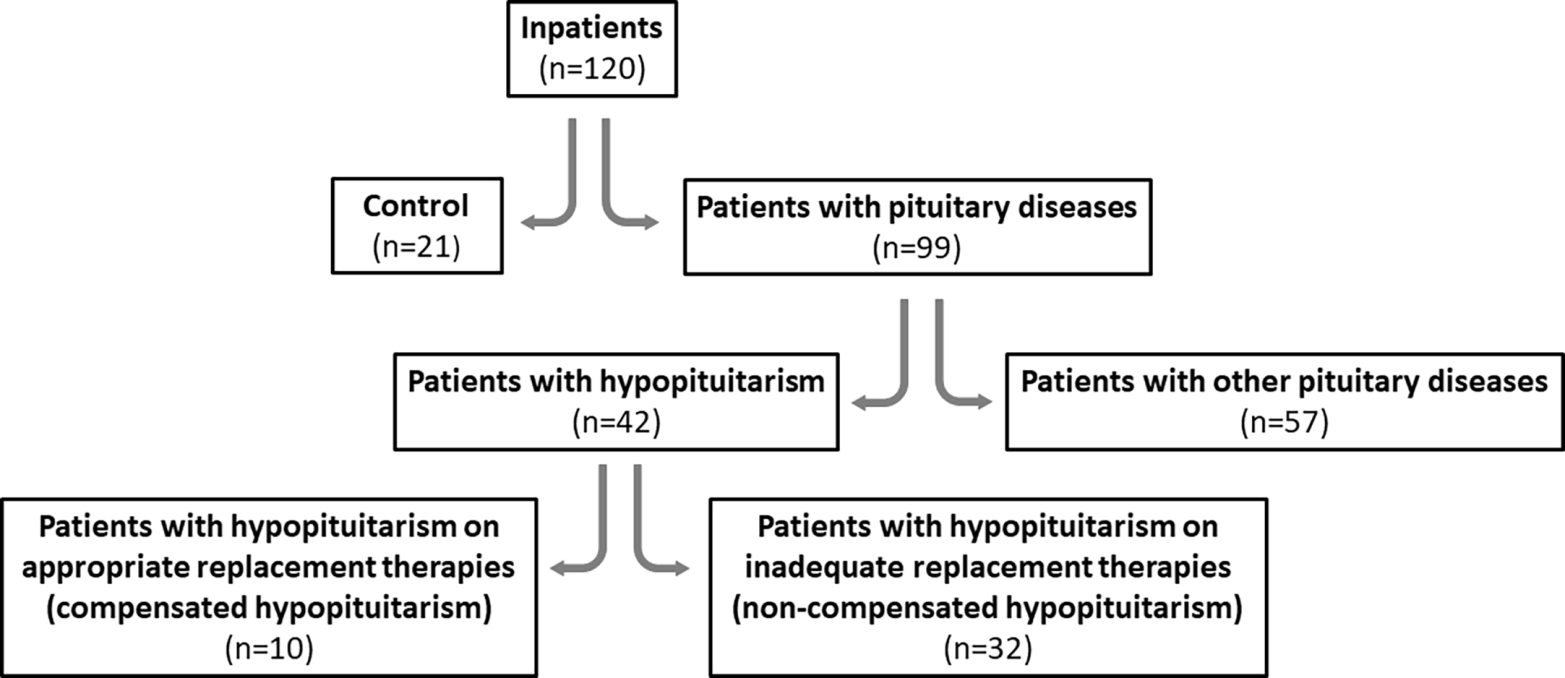
Study design diagram.

Study design diagram is presented in **Figure 1**.

Exclusion criteria constituted: malignant diseases and severe acute/chronic diseases other than pituitary or thyroid diseases.

Body mass and body height were measured to calculate BMI.

Blood samples were collected after an overnight fast at 7 a.m. and all laboratory parameters were measured either in the whole blood or blood serum for diagnostic purposes. Additionally, blood sample (1 mL) was centrifuged (3000 x g, 10 min, 4°C) in order to obtain serum, and stored at -80°C until appropriate assay to measure MBL level.

### 2.1 Parameters measured in blood serum

The concentrations of hormones (thyroid-stimulating hormone, TSH; free triiodothyronine, FT3; free thyroxine, FT4; luteinizing hormone, LH; follicle-stimulating hormone, FSH; adrenocorticotropic hormone; ACTH; cortisol; growth hormone, GH; prolactin, Prl; parathyroid hormone, PTH), of thyroid antibodies (thyroid peroxidase antibody, TPOAb; thyroglobulin antibody, TgAb; TSH receptor antibody, TSHRAb) and of vitamin D were measured in blood serum with immunochemiluminescent method (Cobas e-601; Roche Diagnostics). Other laboratory parameters (insulin-like growth factor I, IGF-I; total cholesterol, TChol; HDL cholesterol, HDLC; LDL cholesterol, LDLC; triglycerides, TGs; glucose; aspartate aminotransferase, ASPAT; alanine aminotransferase, ALAT; bilirubin; sodium, Na; potassium, K; chlorides, Cl; calcium, Ca; urea; creatinine; C-reactive protein, CRP) were measured in blood serum with standard methods (Vitros 4600/Vitros 5.1; Johnson&Johnson). Complete blood count, *i.e.*, red blood cells, RBC; hemoglobin, Hgb; white blood cells, WBC; hematocrit, HCT; platelets; neutrophils; lymphocytes; eosinophils; basophils; and monocytes were measured with Sysmex XN-2000 Hematology System.

Anthropometric measurements included body height and body mass, which were used to calculate Body Mass Index (BMI).

### 2.2 Assay to measure MBL

Serum concentration of MBL was measured by an enzyme-linked immunoabsorbant assay (ELISA) using a commercial *Homo sapiens (Human) MBL ELISA KIT* (Cloud-Clone Corp, USA, no. SEB480Hu), with a detection threshold of 0.156-10 ng/mL. The kit is a sandwich enzyme immunoassay for *in vitro* quantitative measurement of MBL. The protocol for the ELISA was performed following the manufacturer’s guidelines. The absorbance was measured with the use of multifunctional microplate reader (VICTOR X4, Perkin Elmer, USA) with a wavelength of 450 ± 10 nm. Each sample was measured twice. The concentration of the tested factor in the analyzed sample was determined on the basis of a calibration curve created with the use of Four Parameter Logistic (4PL) Regression. WorkOut 2.5 software was used for the analysis.

### 2.3 Statistical analyses

The Student’s unpaired *t* test or Mann-Whitney U test were used to compare differences between independent groups; the results are presented as means ± SEM or median ± SEM, respectively. To evaluate correlations between MBL level and all other linear parameters, Pearson’s correlation coefficient was applied.

Univariate followed by multivariate regression analyses were performed to determine which linear (continuous) variables might have been associated with considered dichotomized variables (patients with hypopituitarism vs. patients without hypopituitarism, compensated vs. non-compensated hypopituitarism).

The 2-sided ratio comparison test was used to determine the probability (frequency) of events.

Distribution of number/percentage of patients with different MBL concentrations is presented on histograms.

Statistical analysis was performed using SigmaPlot version 11.0 (RRID : SCR_003210) (Systat Software, Inc., San Jose, CA, USA). Statistical significance has been determined at the level of *p*<0.05.

Experimental individuals were not randomized into groups because this was deemed irrelevant to this study. Blinding was unnecessary as this is not a randomized controlled trial (and individuals simply could not ethically be blinded).

## 3 Results

All linear laboratory parameters in control individuals (n=21) were compared to those obtained in patients with hypopituitarism (n=42), in patients with other pituitary diseases (n=57) and also in patients with pituitary diseases (n=42+57) (**Table 1**). Patients with hypopituitarism and also all patients with pituitary diseases were, unfortunately, older than control individuals. The groups did not differ regarding body mass and BMI. Expectedly concentrations of some pituitary hormones or hormones released by their target peripheral glands were lower in patients with hypopituitarism (and also in patients with other pituitary diseases or in all patients with pituitary diseases) than in control individuals and these differences relate to ACTH, LH, FSH (of borderline significance), TSH and, regarding peripheral hormones, to cortisol and FT3. Of importance is the finding that IGF-I concentration was significantly lower in patients with hypopituitarism comparing to healthy individuals as well as comparing to patients with other pituitary diseases (*p=*0.019 in case of both comparisons). Regarding lipid profile, total cholesterol was higher in patients with hypopituitarism than in controls (*p=*0.043).

**Table 1:** Clinical/laboratory parameters in control individuals (n=21) vs. patients with hypopituitarism (n=42), vs. patients with other pituitary diseases (n=57) and vs. all patients with pituitary diseases (n=99).

	Control (n=21)	Patients with hypopituitarism (n=42)	Patients with other pituitary diseases (n=57)	Patients with pituitary diseases (n=99)
**Age [years]**	37.92 ± 3.77 n=21	50.26 ± 2.79 n=42 *p=*0.012	45.47 ± 2.21 n=57 *p=*0.084	47.50 ± 1.75 n=99 *p=*0.024
**Body mass [kg]**	75.94 ± 7.66 n=18	79.21 ± 3.05 n=42 *p=*0.640	79.45 ± 2.42 n=50 *p=*0.567	79.35 ± 7.66 n=84 *p=*0.527
**Height [cm]**	167.64 ± 2.25 n=18	162.71 ± 4.28 n=42 *p=*0.425	167.68 ± 1.19 n=50 *p=*0.986	165.68 ± 1.85 n=84 *p=*0.639
**BMI [kg/m^2^]**	26.83 ± 2.53 n=18	27.70 ± 0.79 n=34 *p=*0.682	28.24 ± 0.82 n=18 *p=*0.489	28.02 ± 0.58 n=84 *p=*0.485
**ACTH [pg/mL]**	44.81 ± 5.51 n=16	26.98 ± 4.33 n=34 *p=*0.019	30.87 ± 2.68 n=53 *p=*0.018	29.35 ± 2.35 n=87 *p=*0.011
**LH [IU/L]**	14.00 ± 3.11 n=20	4.19 ± 0.89 n=37 *p*<0.001	13.40 ± 1.62 n=53 *p=*0.854	9.51 ± 1.12 n= 91 *p=*0.111
**FSH [IU/L]**	15.47 ± 4.66 n=20	7.18 ± 1.81 n=36 *p=*0.054	22.28 ± 3.38 n=54 *p=*0.279	16.06 ± 2.26 n=91 *p=*0.912
**Prl [ng/mL]**	14.33 ± 1.88 n=21	305.44 ± 226.75 n=39 *p=*0.352	40.06 ± 10.50 n=53 *p=*0.130	152.56 ± 96.58 n=92 *p=*0.497
**IGF-I [ng/mL]**	190.04 ± 19.17 n=18	114.85 ± 19.03 n=40 *p=*0.019*	184.41 ± 20.93 n=51 *p=*0.880	153.84 ± 14.9 n=91 *p=*0.295
**Cortisol [µg/L]** **(µg/dL)**	157.8 ± 11.9 (15.78 ± 1.19) n=20	78.8 ± 10.6 (7.88 ± 1.06) n=42 *p*<0.001	134.2 ± 5.2 (13.42 ± 0.52) n=56 *p=*0.040	110.4 ± 6.1 (11.04 ± 0.61) n=98 *p=*0.001
**TSH [mIU/L]**	2.39 ± 0.21 n=21	1.20 ± 0.21 n=40 *p*<0.001	1.77 ± 0.14 n=57 *p=*0.022	1.54 ± 0.12 n=99 *p=*0.003
**FT3 [pg/mL]**	3.16 ± 0.11 n=21	2.43 ± 0.08 n=42 *p*<0.001	2.69 ± 0.05 n=57 *p*<0.001	2.58 ± 0.05 n=99 *p*<0.001
**FT4 [ng/L]** **(ng/dL)**	12.4 ± 0.4 (1.24 ± 0.04) n=21	11.6 ± 0.6 (1.16 ± 0.06) n=42 *p=*0.399	12.3 ± 0.2 (1.23 ± 0.02) n=57 *p=*0.811	12.0 ± 0.3 (1.20 ± 0.03) n=99 *p=*0.560
**TPOAb [IU/mL]**	42.69 ± 29.87 n=18	31.30 ± 10.12 n=27 *p=*0.678	43.06 ± 12.81 n=40 *p=*0.990	38.32 ± 8.64 n= 67 *p=*0.847
**TgAb [IU/mL]**	57.61 ± 29.59 n=18	23.73 ± 6.09 n=25 *p=*0.200	156.77 ± 91.07 n=39 *p=*0.470	101.93 ± 54.19 n= 66 *p=*0.675
**TSHRAb [IU/L**	0.84 ± 0.09 n=18	0.86 ± 0.07 n=24 *p=*0.832	0.68 ± 0.06 n=39 *p=*0.155	0.74 ± 0.04 n= 65 *p=*0.325
**TChol [mg/L]** **(mg/dL)**	1713.3 ± 72.1 (171.33 ± 7.21) n=21	1960.2 ± 76.2 (196.02 ± 7.62) n=42 *p=*0.043	1814.6 ± 57.2 (181.46 ± 5.72) n=57 *p=*0.334	1876.4 ± 46.5 (187.64 ± 4.65) n=99 *p=*0.128
**HDLC [mg/L]** **(mg/dL)**	501.9 ± 28.1 (50.19 ± 2.81) n=21	521.4 ± 24.0 (52.14 ± 2.40) n=42 *p=*0.622	514.6 ± 20.6 (51.46 ± 2.06) n=57 *p=*0.741	517.5 ± 15.6 (51.75 ± 1.56) n=99 *p=*0.668
**LDLC [mg/L]** **(mg/dL)**	953.8 ± 52.2 (95.38 ± 5.22) n=21	1071.4 ± 59.7 (107.14 ± 5.97) n=42 *p=*0.208	1054.9 ± 42.9 (105.49 ± 4.29) n=57 *p=*0.196	1061.9 ± 35.2 (106.19 ± 3.52) n=99 *p=*0.181
**HDLC/T.Chol.**	0.295 ± 0.02 n=21	0.284 ± 0.02 n=42 *p=*0.696	0.29 ± 0.01 n=56 *p=*0.964	0.29 ± 0.01 n=98 *p=*0.873
**TGs [mg/L]** **(mg/dL)**	1425.7 ± 197.9 (142.57 ± 19.79) n=21	1549.5 ± 141.4 (154.95 ± 14.14) n=42 *p=*0.624	1367.5 ± 97.6 (136.75 ± 9.76) n=57 *p=*0.772	1443.2 ± 82.3 (144.32 ± 8.23) n=99 *p=*0.930
**Glucose [mg/L]** **(mg/dL)**	891.9 ± 32.4 (89.19 ± 3.24) n=21	909 ± 40.5 (90.90 ± 4.05) n=42 *p=*0.783	897.4 ± 19.1 (89.74 ± 1.91) n=57 *p=*0.883	902.3 ± 20.3 (90.23 ± 2.03) n=99 *p=*0.824
**RBC [10^12^/L]**	4.59 ± 0.12 n=21	4.37 ± 0.08 n=42 *p=*0.133	4.44 ± 0.06 n=57 *p=*0.254	4.41 ± 0.05 n=99 *p=*0.151
**Hgb [g/L]** **[g/dL]**	138.2 ± 4.2 (13.82 ± 0.42) n=21	128.5 ± 3.6 (12.85 ± 0.36) n=42 *p=*0.107	130.6 ± 1.9 (13.06 ± 0.19) n=57 *p=*0.059	129.7 ± 1.9 (12.97 ± 0.19) n=99 *p=*0.061
**WBC [10^9^/L]**	6.39 ± 0.36 n=21	7.21 ± 0.31 n=42 *p=*0.114	6.63 ± 0.29 n=57 *p=*0.655	6.87 ± 0.21 n=99 *p=*0.330
**HCT [vol%]**	40.30 ± 1.14 n=21	38.15 ± 0.60 n=42 *p=*0.071	38.12 ± 0.58 n= 57 *p=*0.068	38.13 ± 0.42 n=99 *p=*0.041
**Platelets[10^9^/L]**	220.91 ± 8.73 n=21	227.29 ± 8.72 n=42 *p=*0.646	241.33 ± 8.08 n=57 *p=*0.159	235.37 ± 5.96 n=99 *p=*0.288
**Neutrophils** **[10^9^/L]**	3.43 ± 0.30 n=21	3.33 ± 0.21 n=42 *p=*0.788	3.49 ± 0.19 n=57 *p=*0.866	3.42 ± 0.14 n=99 *p=*0.984
**Lymphocytes** **[10^9^/L]**	2.241 ± 0.12 n=21	2.87 ± 0.19 n=42 *p=*0.035	2.32 ± 0.12 n=57 *p=*0.693	2.55 ± 0.11 n=99 *p=*0.207
**Eosinophils** **[10^9^/L]**	0.178 ± 0.02 n=21	0.247 ± 0.05 n=42 *p=*0.354	0.21 ± 0.02 n=57 *p=*0.396	0.22 ± 0.02 n=99 *p=*0.389
**Basophils** **[10^9^/L]**	0.040 ± 0.003 n=21	0.051 ± 0.007 n=42 *p=*0.317	0.040 ± 0.003 n=57 *p=*0.993	0.045 ± 0.004 n= 99 *p=*0.569
**Monocytes** **[10^9^/L]**	0.507 ± 0.03 n=21	0.617 ± 0.052 n=42 *p=*0.163	0.59 ± 0.03 n=57 *p=*0.173	0.60 ± 0.03 n=99 *p=*0.155
**ASPAT [U/L]**	31.09 ± 2.31 n=21	27.83 ± 1.92 n=41 *p=*0.305	24.29 ± 0.93 n=57 *p=*0.002	25.78 ± 0.98 n=98 *p=*0.027
**ALAT [U/L]**	30.60 ± 3.96 n=20	25.72 ± 1.92 n=40 *p=*0.216	22.11 ± 1.48 n=56 *p=*0.015	23.61 ± 1.19 n=96 *p=*0.029
**Bilirubin [mg/L]** **(mg/dL])**	8.42 ± 1.1 (0.842 ± 0.11) n=17	7.7 ± 0.8 (0.77 ± 0.08) n=38 *p=*0.593	7.2 ± 0.4 (0.72 ± 0.04) n=53 *p=*0.234	7.4 ± 0.4 (0.74 ± 0.04) n=91 *p=*0.347
**Na [mmol/L]**	139.90 ± 0.43 n=21	139.67 ± 0.46 n=42 *p=*0.742	139.72 ± 0.29 n=57 *p=*0.737	139.70 ± 0.26 n=99 *p=*0.727
**K [mmol/L]**	4.27 ± 0.06 n=21	5.19 ± 0.92 n=42 *p=*0.483	4.23 ± 0.04 n=57 *p=*0.630	4.64 ± 0.39 n=99 *p=*0.667
**Cl [mmol/L]**	104.62 ± 0.66 n=21	104.14 ± 0.59 n=41 *p=*0.623	105.61 ± 0.36 n=57 *p=*0.164	105.00 ± 0.33 n=98 *p=*0.624
**Ca [mmol/L]**	2.31 ± 0.03 n=19	2.29 ± 0.021 n=36 *p=*0.559	2.27 ± 0.02 n=50 *p=*0.251	2.28 ± 0.01 n=86 *p=*0.331
**Urea [mg/L]** **(mg/dL)**	311.9 ± 25.8 (31.19 ± 2.58) n=16	330.3 ± 15.1 (33.03 ± 1.51) n=39 *p=*0.526	317.1 ± 17.3 (31.71 ± 1.73) n=49 *p=*0.876	322.9 ± 11.7 (32.29 ± 1.17) n=88 *p=*0.708
**Creatinine [mg/L]** **(mg/dL)**	7.53 ± 0.3 (0.753 ± 0.03) n=20	8.34 ± 0.3 (0.834 ± 0.03) n=42 *p=*0.105	7.4 ± 0.3 (0.74 ± 0.03) n=56 *p=*0.761	7.8 ± 0.2 (0.78 ± 0.02) n=98 *p=*0.626
**GFR [mL/(min×1.72 m²)]**	97.52 ± 4.32 n=20	89.70 ± 6.58 n=42 *p=*0.438	99.56 ± 3.81 n=56 *p=*0.768	95.33 ± 3.58 n=98 *p=*0.790
**Vit D [ng/mL]**	25.70 ± 2.41 n=16	29.92 ± 2.18 n=33 *p=*0.242	26.86 ± 2.04 n=39 *p=*0.745	28.26 ± 1.49 n=72 *p=*0.448
**PTH [pg/mL]**	35.16 ± 3.92 n=16	42.72 ± 3.27 n=35 *p=*0.177	53.94 ± 11.49 n=44 *p=*0.335	48.97 ± 6.56 n=79 *p=*0.351

Comparison between subgroups was performed by Student’s unpaired *t*-test. Statistical significance was determined at the level of *p*<0.05. *p=0.019 vs. Control and vs. patients with other pituitary diseases. Statistically significant differences are shaded.

Because MBL level can be affected by inflammation, we have checked the prevalence of the increased C reactive protein (CRP) concentrations in particular groups and we did not find statistically significant differences between any groups, *i.e.* control (1/15, 7%) vs. patients with hypopituitarism (7/33, 21%), *p=*0.233; control (1/15, 7%) vs. patients with other pituitary diseases (6/45, 13%), *p=*0.531; patients with hypopituitarism (7/33, 21%) vs. patients with other pituitary diseases (6/45, 13%), *p=*0.349. In control individuals only one patient had slightly increased CRP level, *i.e.* 14.8 mg/L (1.48 mg/dL), comparing to the upper normal range being 10 mg/L (1 mg/dL). In hypopituitary patients and in patients with other pituitary diseases the highest CRP levels were 14.2 mg/L (1.42 mg/dL) and 14.9 mg/L (1.49 mg/dL), respectively (data not shown).

**Figure 2:**
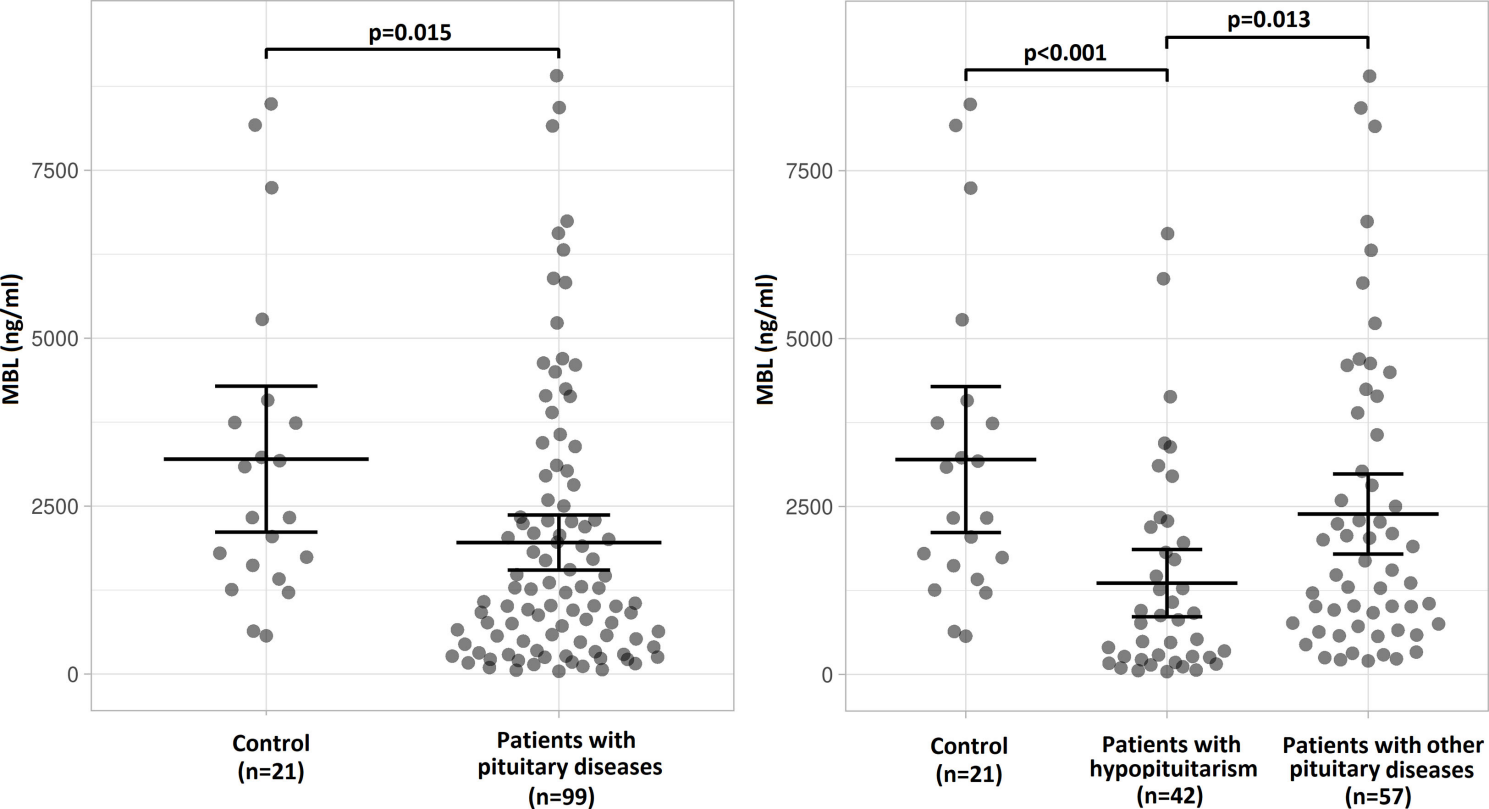
Mean (± SEM) values of mannan-binding lectin (MBL) level in control individuals (n=21) and in patients with pituitary diseases (n=99) (left) or mean (± SEM) values of MBL level in control individuals (n=21), in patients with hypopituitarism (n=42) and in patients with other pituitary diseases (n=57) (right). Statistical evaluation was performed by an unpaired Student’s *t*-test. The statistical differences are marked in the graphics.

The concentration of MBL in blood serum was significantly lower in patients with pituitary diseases (n=99) than in control individuals (**Figure 2** left). When patients with hypopituitarism (n=42) and patients with other pituitary diseases (n=57) were considered separately, the lowest MBL level was found in patients with hypopituitarism – comparing to control individuals, as well as comparing to patients with other pituitary diseases (**Figure 2** right).

**Figure 3:**
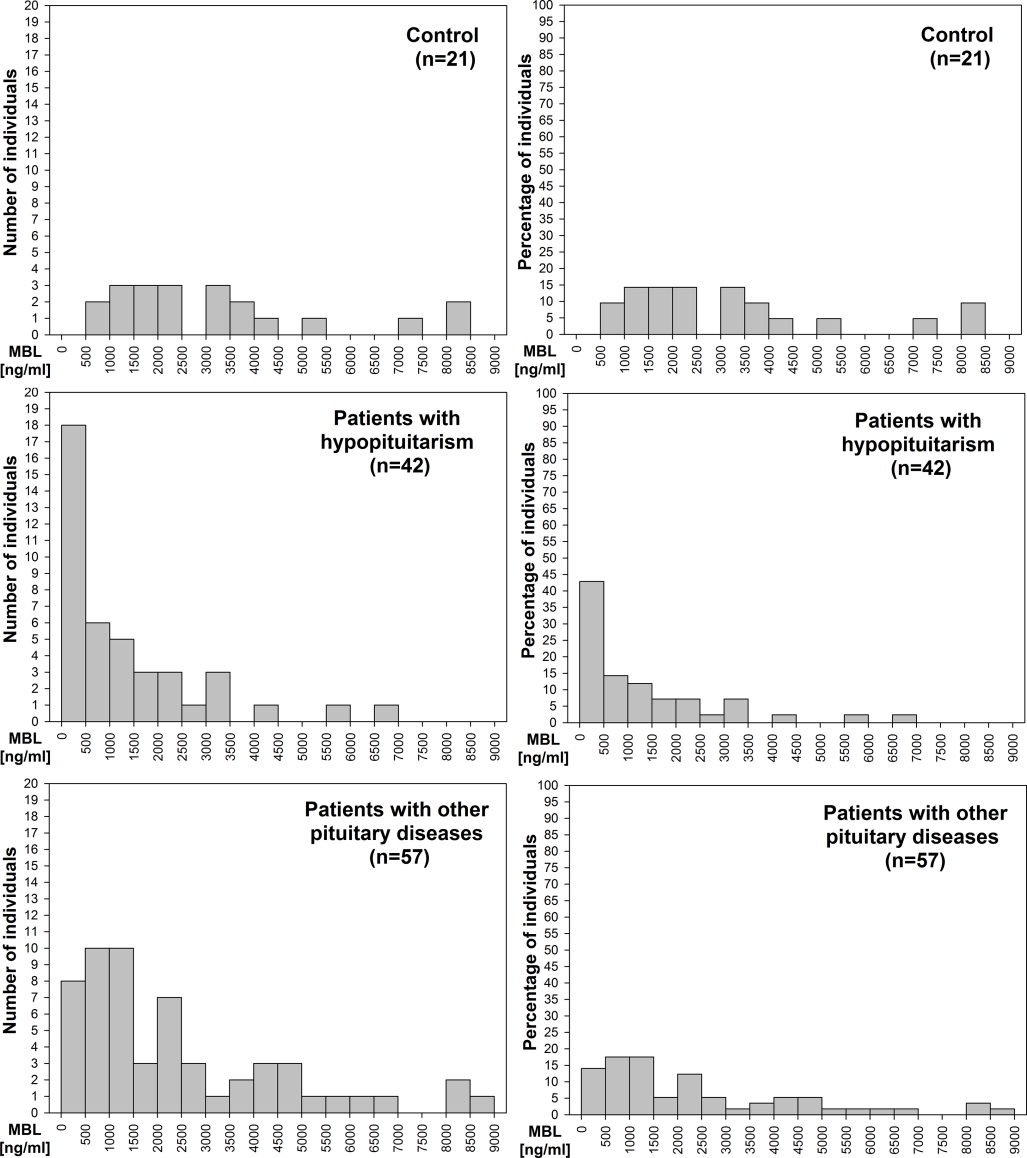
Histograms of the distribution of number of patients (left graphs) or percentage of patients (right graphs) with relation to mannan-binding lectin (MBL) level in control individuals (n=21), in patients with hypopituitarism (n=42), and in patients with other pituitary diseases (n=57).

We evaluated the distribution of patients (number of patients – **Figure 3** left graphs; percentage of patients – **Figure 3** right graphs) with relation to MBL level. There is a clear difference in this distribution between control individuals, in whom no subjects had MBL level below 500 ng/mL and most subjects, i.e. 19/21 (90.5%), had MBL level above 1000 ng/mL, *versus* hypopituitary patients, in whom as many as 18/42 patients (42.9%) had MBL level below 500 ng/mL and 18/42 patients (42.9%) had MBL level above 1000 ng/mL. Histogram presenting patients with other pituitary diseases is something in between the two, with only 8/57 patients (14%) having MBL concentration below 500 ng/mL, and with as many as 39/57 patients (68.4%) who had MBL level above 1000 ng/mL.

In the group of patients with hypopituitarism (n=42) we have checked if patients with the lowest levels of MBL (<500 ng/mL) differ from patients with higher levels of MBL (>500 ng/mL) regarding their age. The mean age of patients with MBL levels <500 ng/mL (n=18) was 49.39± 4.44 whereas of those with MBL levels above this cut-off point (n=24) the mean age was 50.92 ± 3.65 and this difference was not statistically significant (*p=*0.790). Similarly, no differences regarding mean age of patients with lower levels of MBL (<500 ng/mL) (n=13) *versus* higher levels of MBL (>500 ng/mL) (n=44) were found when the group of patients with other pituitary diseases (n=57) was considered (48.38 ± 4.11 vs. 44.61 ± 2.60, *p=*0.479).

**Figure 4:**
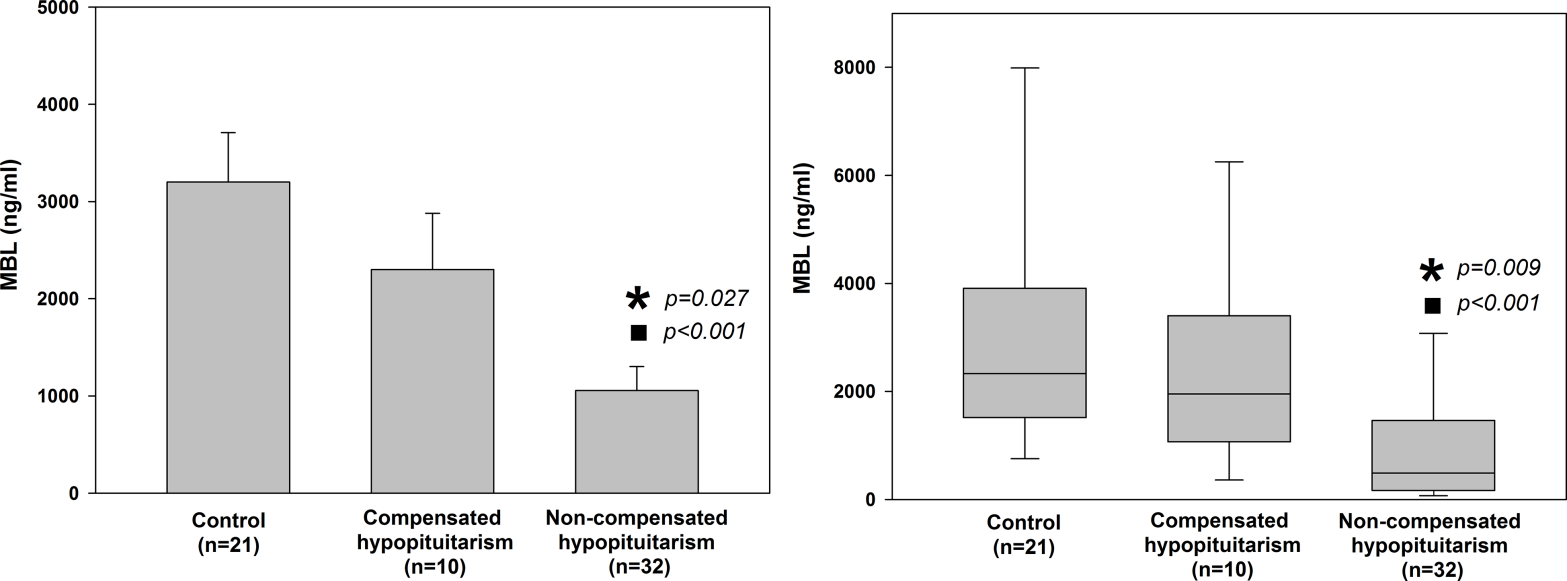
Bar chart of mean (± SEM) (left graph) and box plot of median (± SEM) (right graph) values of mannan-binding lectin (MBL) level in control individuals (n=21) and patients with hypopituitarism (n=42) who are on appropriate replacement therapies (compensated hypopituitarism, n=10) vs. inadequate replacement therapies (non-compensated hypopituitarism, n=32). Central line of each box denotes the median, the top and bottom edges of the box show the 25% and 75% percentile, with the 10% and 90% percentiles shown by the upper and lower whiskers. Statistical evaluation was performed by an unpaired Student’s *t*-test or Mann-Whitney U test respectively. *p<0.05 vs. compensated hypopituitarism, ^◼^ *p*<0.05 vs control individuals.

We compared mean and median values of MBL level in a group of patients with hypopituitarism who were on appropriate replacement therapies (compensated hypopituitarism) *versus* patients being on inadequate replacement therapies (non-compensated hypopituitarism) (**Figure 4**). Patients with non-compensated hypopituitarism did have lower mean MBL level (**Figure 4** left graph) and lower median MBL level (**Figure 4** right graph) comparing to patients with compensated hypopituitarism. Patients with non-compensated hypopituitarism obviously had lower mean MBL level (**Figure 4** left graph) and lower median MBL level (**Figure 4** right graph) comparing to control individuals. However, mean and median MBL levels in patients with compensated hypopituitarism *versus* controls did not differ significantly (**Figure 4**, 2300.09 ± 579.93 vs. 3199.30 ± 508.46, *p=*0.294; 1951.90 vs. 2329.16; *p=*0.301, respectively).

**Figure 5:**
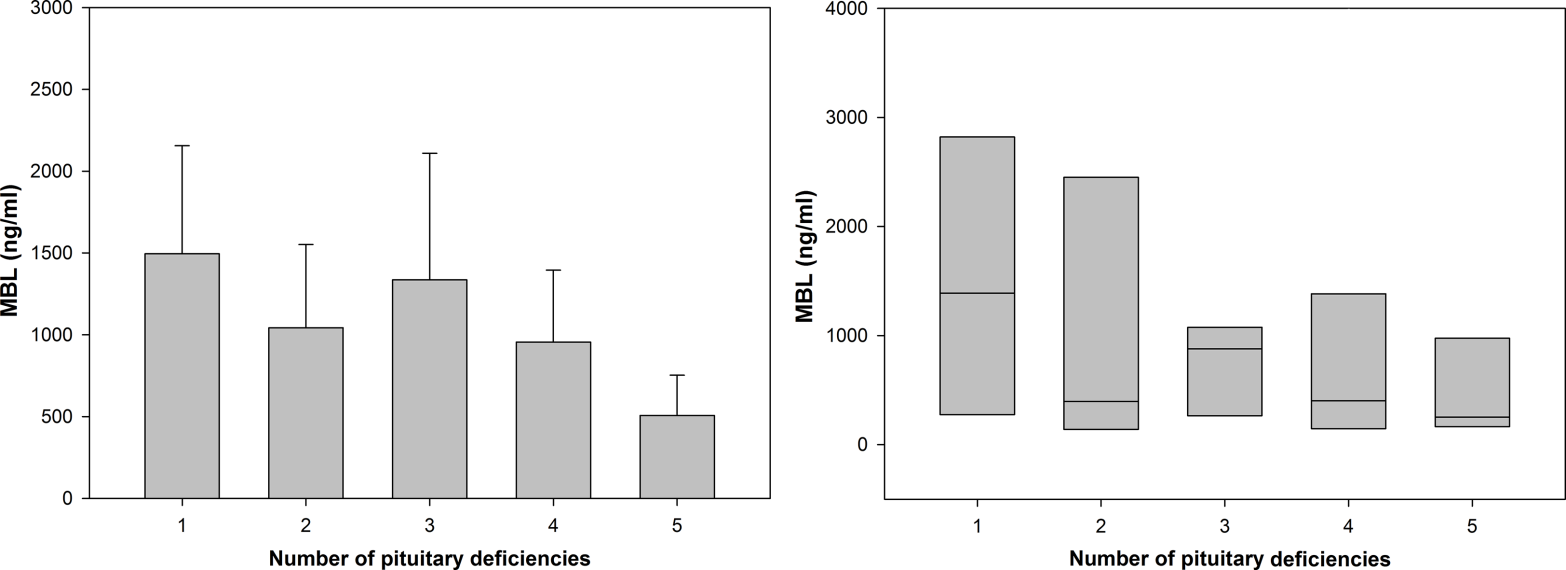
Bar chart of mean (± SEM) (left graph) and box plot of median (± SEM) (right graph) values of mannan-binding lectin (MBL) level in patients with hypopituitarism who are on inadequate replacement therapies (non-compensated hypopituitarism) presented in particular subgroups of individuals depending on how many pituitary axes are involved. Central line of each box denotes the median, the top and bottom edges of the box show the 25% and 75% percentile. No statistically significant differences were found.

Additionally, MBL level was compared between patients with compensated *versus* non-compensated hypopituitarism depending on how many pituitary axes were involved, *i.e.* on the number of pituitary deficiencies (**Table 2**). Although no statistically significant differences were found (due to low numbers of individuals and to very high standard error of the mean, SEM) mean MBL levels tend to be higher in patients with compensated hypopituitarism in most of subgroups considered. Of importance is the finding that among patients with non-compensated hypopituitarism a tendency is seen for lower mean (**Figure 5** left) and median (**Figure 5** right) MBL levels in patients with higher numbers of pituitary deficiencies; the lowest MBL level was found in patients with panhypopituitarism. Unfortunately, these differences did not reach statistical significance (**Table 2** and **Figure 5**).

**Table 2:** Mean (± SEM) values of mannan-binding lectin (MBL) level in patients with hypopituitarism (n=42) who are on appropriate replacement therapies (compensated hypopituitarism, n=10) vs. inadequate replacement therapies (non-compensated hypopituitarism, n=32) presented in particular subgroups of individuals depending on how many pituitary axes are involved.

	Compensated hypopituitarism (n=10)	Non-compensated hypopituitarism (n=32)
One pituitary deficiency (n=9)	1662.73 ± 581.48 n=5	1494.68 ± 660.73 n=4
Two pituitary deficiencies (n=7)	6562.51 n=1	1042.48 ± 509.37 n=6
Three pituitary deficiencies (n=9)	1736.58 ± 456.18 n=2	1335.99 ± 773.59 n=7
Four pituitary deficiencies (n=12)	2325.82 ± 1061.62 n=2	955.13 ± 439.57 n=10
Five pituitary deficiencies (n=5)	- n=0	507.02 ± 246.51 n=5

Statistical evaluation was performed by Mann-Whitney U test. No statistically significant differences were found.

Possible correlations between MBL level and all other linear parameters were evaluated in control individuals, in patients with hypopituitarism, in patients with other pituitary diseases, and it the whole group of individuals (**Table 3**). Of great importance with relation to the findings of the present study is the observation that MBL level did not correlate with age in any of the groups considered. The most prominent result does constitute the positive correlation between MBL level and insulin-like growth factor I (IGF-I) level in patients with hypopituitarism and in patients with other pituitary diseases and also in the whole group of individuals. Positive correlation between MBL level and glucose concentration was found in patients with hypopituitarism. MBL level also correlated with FT3 concentration in the whole group examined. Apart from patients with hypopituitarism, in all other groups MBL level correlated with hematocrit (HCT) as well as with red blood cells (RBC) (the latter with borderline significance). Other correlations such as between MBL level and TgAb concentration or hemoglobin (Hgb) in controls or between MBL level and bilirubin concentration in patients with hypopituitarism or between MBL level and potassium concentration (negative correlation) in patients with other pituitary diseases can be neglected and therefore they will be not discussed in this paper.

**Table 3:** Correlations between mannan-binding lectin (MBL) level and clinical/laboratory parameters in 4 groups considered: Control individuals (n=21), patients with hypopituitarism (n=42), patients with other pituitary diseases (n=57), and the whole group of individuals (n=120).

	MBL [ng/mL] r; *p*-value; n			
	Control individuals (n=21)	Patients with hypopituitarism (n=42)	Patients with other pituitary diseases (n=57)	The whole group of individuals (n=120)
**Age [years]**	0.0149 *p=*0.949 n=21	-0.1050 *p=*0.512 n=42	-0.0404 *p=*0.765 n=57	-0.115 *p=*0.213 n=120
**Body mass [kg]**	-0.0740 *p=*0.770 n=18	0.1010 *p=*0.576 n=33	-0.0142 *p=*0.922 n=50	-0.0192 *p=*0.849 n=101
**Height [cm]**	0.305 *p=*0.219 n=18	-0.297 *p=*0.0932 n=33	-0.0505 *p=*0.728 n=50	-0.0271 *p=*0.788 n=101
**BMI [kg/m^2^]**	-0.163 *p=*0.519 n=18	0.0802 *p=*0.657 n=33	0.0121 *p=*0.934 n=50	-0.0381 *p=*0.706 n=101
**ACTH [pg/mL]**	-0.142 *p=*0.599 n=16	-0.0887 *p=*0.618 n=34	-0.124 *p=*0.375 n=53	-0.0373 *p=*0.709 n=103
**LH [IU/L]**	0.0287 *p=*0.904 n=20	0.0260 *p=*0.880 n=36	0.102 *p=*0.467 n=53	0.121 *p=*0.207 n=110
**FSH [IU/L]**	-0.0815 *p=*0.733 n=20	0.0036 *p=*0.983 n=36	-0.0922 *p=*0.503 n=55	-0.0159 *p=*0.869 n=111
**Prl [ng/mL]**	0.161 *p=*0.485 n=21	-0.0805 *p=*0.631 n=38	-0.0174 *p=*0.902 n=53	-0.0821 *p=*0.390 n=112
**IGF-I [ng/mL]**	0.220 *p=*0.381 n=18	0.4410 *p=*0.0049 n=40	0.429 *p=*0.002 n=51	0.434 *p*<0.001 n=108
**Cortisol [µg/L]**	0.120 *p=*0.614 n=20	0.0788 *p=*0.620 n=40	-0.173 *p=*0.203 n=56	0.141 *p=*0.127 n=116
**TSH [mIU/L]**	0.132 *p=*0.569 n=21	0.1630 *p=*0.320 n=39	-0.0464 *p=*0.732 n=57	0.144 *p=*0.122 n=117
**FT3 [pg/mL]**	0.122 *p=*0.599 n=21	0.2920 *p=*0.0639 n=42	0.149 *p=*0.268 n=57	0.294 *p=*0.0012 n=120
**FT4 [ng/L]**	0.223 *p=*0.332 n=21	-0.190 *p=*0.235 n=42	-0.133 *p=*0.324 n=57	-0.0562 *p=*0.543 n=120
**TPOAb [IU/mL]**	0.190 *p=*0.450 n=18	-0.200 *p=*0.317 n=27	-0.193 *p=*0.234 n=40	-0.0305 *p=*0.782 n=85
**TgAb [IU/mL]**	0.560 *p=*0.0156 n=18	-0.0758 *p=*0.707 n=27	-0.160 *p=*0.332 n=39	-0.0487 *p=*0.660 n=84
**TSHRAb [IU/L**	-0.289 *p=*0.246 n=18	0.1960 *p=*0.337 n=26	0.202 *p=*0.217 n=39	0.0581 *p=*0.602 n=83
**TChol [mg/L]**	-0.209 *p=*0.363 n=21	-0.0858 *p=*0.594 n=42	0.135 *p=*0.316 n=57	-0.0441 *p=*0.634 n=120
**HDLC [mg/L]**	-0.0313 *p=*0.893 n=21	-0.2720 *p=*0.0854 n=42	0.0268 *p=*0.843 n=57	-0.0768 *p=*0.407 n=120
**LDLC [mg/L]**	-0.191 *p=*0.408 n=21	-0.0236 *p=*0.884 n=42	0.132 *p=*0.326 n=57	0.00642 *p=*0.945 n=120
**HDLC/TChol**	-0.0077 *p=*0.973 n=21	-0.2060 *p=*0.196 n=42	-0.138 *p=*0.312 n=56	-0.111 *p=*0.229 n=118
**TGs [mg/L]**	-0.143 *p=*0.535 n=21	-0.1680 *p=*0.292 n=42	-0.160 *p=*0.332 n=39	-0.0309 *p=*0.739 n=120
**Glucose [mg/L]**	-0.00914 *p=*0.694 n=21	0.3670 *p=*0.0182 n=42	0.207 *p=*0.122 n=57	0.177 *p=*0.0537 n=120
**RBC [10^12^/L]**	0.425 *p=*0.054 n=21	-0.1550 *p=*0.332 n=42	0.248 *p=*0.0633 n=57	0.204 *p=*0.0264 n=119
**Hgb [g/L]**	0.575 *p=*0.006 n=21	-0.0836 *p=*0.603 n=42	0.203 *p=*0.130 n=57	0.0793 *p=*0.391 n=120
**WBC [10^9^/L]**	-0.259 *p=*0.257 n=21	-0.2860 *p=*0.0698 n=42	0.0616 *p=*0.649 n=57	-0.124 *p=*0.179 n=120
**HCT [vol%]**	0.500 *p=*0.0209 n=21	-0.0961 *p=*0.550 n=42	0.263 *p=*0.0481 n=57	0.259 *p=*0.004 n=120
**Platelets [10^9^/L]**	-0.315 *p=* 0.165 n=21	-0.2920 *p=* 0.0638 n=42	-0.0641 *p=*0.636 n=57	-0.149 *p=*0.107 n=120
**Neutrophils** **[10^9^/L]**	-0.255 *p=* 0.264 n=21	-0.2480 *p=* 0.118 n=42	0.0788 *p=*0.560 n=57	-0.0564 *p=*0.542 n=120
**Lymphocytes** **[10^9^/L]**	-0.155 *p=* 0.502 n=21	-0.0877 *p=* 0.586 n=42	0.0137 *p=*0.919 n=57	-0.113 *p=*0.222 n=120
**Eosinophils** **[10^9^/L]**	0.183 *p=* 0.426 n=21	-0.0633 *p=* 0.694 n=42	0.0377 *p=*0.780 n=57	-0.0293 *p=*0.751 n=120
**Basophils** **[10^9^/L]**	-0.0394 *p=* 0.865 n=21	-0.1180 *p=* 0.462 n=42	-0.00193 *p=*0.989 n=57	-0.0893 *p=*0.334 n=120
**Monocytes** **[10^9^/L]**	-0.0722 *p=*0.756 n=21	-0.0812 *p=*0.614 n=42	0.104 *p=*0.440 n=57	-0.0214 *p=*0.818 n=120
**ASPAT [U/L]**	0.0158 *p=* 0.946 n=21	-0.0176 *p=* 0.914 n=40	-0.113 *p=*0.402 n=57	-0.0302 *p=*0.746 n=118
**ALAT [U/L]**	-0.0484 *p=* 0.840 n=20	0.0336 *p=* 0.839 n=39	-0.129 *p=*0.342 n=56	-0.0437 *p=*0.643 n=115
**Bilirubin [mg/L]**	0.196 *p=* 0.451 n=17	0.3960 *p=* 0.0154 n=37	-0.0748 *p=*0.594 n=53	0.129 *p=*0.184 n=107
**Na [mmol/L]**	0.411 *p=* 0.064 n=21	0.0700 *p=* 0.664 n=42	-0.0952 *p=*0.481 n=57	0.0481 *p=*0.603 n=120
**K [mmol/L]**	0.363 *p=*0.106 n=21	-0.0645 *p=*0.689 n=42	-0.268 *p=*0.044 n=57	-0.070 *p=*0.449 n=120
**Cl [mmol/L]**	0.290 *p=*0.203 n=21	-0.0422 *p=*0.796 n=41	0.0192 *p=*0.887 n=57	0.0898 *p=*0.334 n=118
**Ca [mmol/L]**	0.158 *p=*0.518 n=19	-0.2630 *p=*0.127 n=35	-0.182 *p=*0.207 n=50	-0.121 *p=*0.220 n=104
**Urea [mg/L]**	0.114 *p=*0.673 n=16	-0.1900 *p=*0.254 n=38	0.0355 *p=*0.809 n=49	-0.0304 *p=*0.761 n=103
**Creatinine [mg/L]**	0.378 *p=*0.100 n=20	-0.1970 *p=*0.217 n=42	0.0394 *p=*0.773 n=56	-0.0272 *p=*0.771 n=117
**GFR [mL/(min×1.72 m²)]**	-0.216 *p=*0.360 n=20	0.2320 *p=*0.144 n=42	0.107 *p=*0.431 n=56	0.131 *p=*0.159 n=118
**Vit D [ng/mL]**	0.335 *p=*0.204 n=16	-0.1410 *p=*0.440 n=32	-0.124 *p=*0.453 n=39	-0.0579 *p=*0.597 n=86
**PTH [pg/mL]**	-0.208 *p=*0.440 n=16	-0.1540 *p=*0.383 n=34	-0.152 *p=*0.324 n=44	-0.124 *p=*0.232 n=94

Statistical evaluation was performed by the Pearson’s correlation test. r - Pearson's correlation coefficient. Statistical significance was determined at the level of *p*<0.05. Statistically significant differences are shaded.

We evaluated the percentage of abnormal lipid profile in control individuals *versus* unhealthy patients (**Table 4**). The increased total cholesterol was found more frequently in patients with hypopituitarism (**Table 4** above) or in patients with pituitary diseases (**Table 4** below) than in control individuals.

**Table 4:** Percentage of abnormal lipid profile in control individuals (n=21) vs. patients with hypopituitarism (n=42) (above), and in control individuals (n=21) vs. patients with pituitary diseases (n=99) (below).

	Control n=21	Patients with hypopituitarism n=42	*p*
**TChol** **≥2000 mg/L** (**≥200 mg/dL)**	n=3 14%	n=18 43%	0.025
**HDLC** **<400 mg/L** **(<40 mg/dL)**	n=5 24%	n=7 17%	0.509
**LDLC** **>1000 mg/L** **(>100 mg/dL)**	n=10 48%	n=23 55%	0.601
**TGs** **>1500 mg/L** **(>150 mg/dL)**	n=7 33%	n=19 45%	0.364
**HDLC/TChol** **<0.2**	n=1 5%	n=3 7%	0.759
	Control n=21	Patients with pituitary diseases n=99	*p*
**TChol** **≥2000 mg/L** (**≥200 mg/dL)**	n=3 14%	n=38 38%	0.037
**HDLC** **<400 mg/L** **(<40 mg/dL)**	n=5 24%	n=17 40%	0.170
**LDLC** **>1000 mg/L** **(>100 mg/dL)**	n=10 48%	n=54 56%	0.504
**TGs** **>1500 mg/L** **(>150 mg/dL)**	n=7 33%	n=40 40%	0.551
**HDLC/TChol** **<0.2**	n=1 5%	n=5 5%	1.000

Statistical evaluation was performed by the two-sided ratio comparison test. Statistical significance was determined at the level of *p*<0.05. Statistically significant differences are shaded.

The univariate/multivariate logistic regression analyses were performed to determine which linear variables are associated with hypopituitarism (**Table 5**). In the univariate analysis conducted in a group of 63 subjects (control individuals plus patients with hypopituitarism; n=21+42) the following three determinants, *i.e.* age, total cholesterol (TChol) concentration and lymphocyte concentration, were positively associated with hypopituitarism, whereas MBL level, TSH concentration, FT3 concentration, LH, ACTH and IGF-I were negatively associated with hypopituitarism. Unfortunately, all these determinants lost their statistical significance in multivariate logistic regression analysis (**Table 5** above).

**Table 5:** Univariate and multivariate logistic regression analyses of **hypopituitarism** determinants (linear variables) performed in the following groups: Control plus patients with hypopituitarism (n=21+42) (above), and the whole group of individuals (n=120) (below).

Variable	Control plus patients with hypopituitarism (n=21+42)					
	Univariate regression			Multivariate regression		
	OR	95%Cl	*p*	OR	95%Cl	*p*
**Age [years]**	1.042	1.01-1.08	*0.017*	*0.922*	*0.62-1.38*	*0.693*
**MBL [ng/mL]**	0.999	0.99-1.00	*0.004*	*0.997*	*0.99-1.01*	*0.519*
**TSH [mIU/L]**	0.455	0.28-0.75	*0.002*	*0.164*	*0.01-26.72*	*0.487*
**FT3 [pg/mL]**	0.043	0.01-0.23	*<0.001*	*<0.001*	*1.53-3.09*	*0.541*
**TChol [mg/L]**	1.013	1.00-1.03	*0.049*	*1.110*	*0.79-1.55*	*0.541*
**LH [IU/L]**	0.878	0.79-0.96	*0.007*	*0.895*	*0.63-1.26*	*0.528*
**ACTH [pg/mL]**	0.970	0.94-0.99	*0.045*	*0.856*	*0.63-1.15*	*0.308*
**IGF-I [ng/mL]**	0.994	0.98-1.00	*0.041*	*1.020*	*0.97-1.07*	*0.441*
**Lymphocytes [10^9^/L]**	1.877	1.01-3.48	*0.046*	*17.272*	*0.04-7195.24*	*0.355*
Variable	Whole group of individuals (n=120)					
	Univariate regression			Multivariate regression		
	OR	95%Cl	*p*	OR	95%Cl	*p*
**Age [years]**	1.023	1.00-1.04	*0.046*	1.031	0.98-1.08	*0.209*
**MBL [ng/mL]**	1.001	0.99-1.00	*0.005*	1.001	0.99-1.00	*0.119*
**TSH [mIU/L]**	0.558	0.38-0.81	*0.002*	0.780	0.45-1.35	*0.377*
**FT3 [pg/mL]**	0.186	0.075-0.46	*<0.001*	0.640	0.13-3.07	*0.577*
**TChol [mg/L]**	1.009	1.00-1.018	*0.045*	1.018	1.00-1.03	*0.019*
**LH [IU/L]**	0.849	0.78-0.93	*<0.001*	0.841	0.68-1.04	*0.115*
**FSH [IU/L]**	0.949	0.91-0.987	*0.008*	1.003	0.91-1.10	*0.952*
**ACTH [pg/mL]**	0.970	0.94-0.99	*0.004*	0.982	0.95-1.01	*0.241*
**IGF-I [ng/mL]**	0.992	0.99-1.00	*0.004*	1.002	0.99-1.01	*0.483*
**Lymphocytes [10^9^/L]**	1.732	1.16-2.58	*0.007*	1.221	0.62-2.41	*0.564*
**Creatinine [mg/L]**	9.108	1.32-62.93	*0.025*	3.227	0.17-59.98	*0.432*

Only statistically significant determinants are presented in the univariate analysis. Statistical significance was determined at the level of *p*<0.05. Statistically significant differences are shaded.

When univariate regression analysis was performed in the whole group of individuals (n=120) the same relationships were found as described above for the group of 63 patients. Additionally, FSH was negatively, whereas creatinine was positively, associated with hypopituitarism. In multivariate regression analysis the only determinant positively associated with hypopituitarism was, unexpectedly, TChol concentration (**Table 5** below).

Another univariate logistic regression analysis was performed only in the group of patients with hypopituitarism to determine which linear variables are associated with inadequate hormone replacement therapies (non-compensated hypopituitarism) (**Table 6**). Three determinants, *i.e.* FT3, LH and MBL levels were negatively associated with inadequate replacement therapies (non-compensated hypopituitarism). In multivariate regression analysis these three determinants lost their statistical significance.

**Table 6:** Univariate logistic regression analysis of **non-compensated hypopituitarism** (inadequate hormone replacement therapies) determinants (linear variables) performed in patients with hypopituitarism (n=42).

Variable	Patients with hypopituitarism (n=42)					
	Univariate regression			Multivariate regression		
	OR	95%Cl	*p*	OR	95%Cl	*p*
**FT3 [pg/mL]**	0.120	0.02-0.2	*0.020*	0.144	0.017-1.18	*0.071*
**LH [IU/L]**	0.814	0.68-0.97	*0.024*	0.800	0.64-0.99	*0.073*
**MBL [ng/mL]**	0.999	0.99-1.00	*0.048*	0.999	0.99-1.00	*0.127*

Only statistically significant determinants are presented in the univariate analysis. Statistical significance was determined at the level of *p*<0.05. Statistically significant differences are shaded.

Because vitamin D deficiency affects the immune system we have checked if the percentages of patients with decreased vitamin D differ between Control and unhealthy individuals and if MBL levels differ between individuals with vitamin D deficiency and with vitamin D sufficiency (**Table 7**). Regarding the first comparison (made with the two-sided ratio comparison test), severe vitamin D deficiency occurred less commonly in patients with hypopituitarism vs. Controls (5/33, 15% vs. 6/16, 37%, *p=*0.087) as well as less commonly in the group of all patients with pituitary diseases (n=99) vs. Control (16/72, 22% vs. 6/16, 37%, *p=*0.211), however these differences did not reach statistical significance (data not presented). Regarding the other comparison, the evaluation was done in the group of patients with hypopituitarism as well as in the whole group examined. Severe vitamin D deficiency <20 ng/mL was characterized, unexpectedly, by higher MBL levels when the evaluation was done in patients with hypopituitarism as well as in the whole group examined, however these differences were of borderline significance (**Table 7** below). The cut-off vitamin D level of 30 ng/mL was not associated with any substantial differences (**Table 7** above).

**Table 7:** Mean MBL concentration in patients with/without Vit. D deficiency (<30 ng/mL/≥30 ng/mL) (above) or severe vitamin D deficiency (<20 ng/mL/≥20 ng/mL) (below) evaluated in control individuals (n=21) and in the whole group of individuals (n=120).

	MBL [ng/mL]		*p*
	Vit D <30 ng/mL	Vit D >30 ng/mL	
**Group of 42 patients**	1528.93 ± 391.49 n=17	1398.11 ± 430.46 n=16	*0.824*
**Group of 120 patients**	2085.88 ± 277.38 n=31	2168.46 ± 415.46 n=57	*0.865*
	MBL [ng/mL]		*p*
	Vit D <20 ng/mL	Vit D >20 ng/mL	
**Group of 42 patients**	2709.24 ± 918.59 n=5	1232.82 ± 280.72 n=27	*0.060*
**Group of 120 patients**	2855.11 ± 513.16 n=21	1911.19 ± 252.19 n=66	*0.080*

Comparison between subgroups was performed by Student’s unpaired *t*-test. Statistical significance was determined at the level of *p*<0.05.

Because the level of thyroid antibodies express thyroid autoimmunity resulting from the disturbed efficiency of the immune system we checked the prevalence of the increased concentrations of thyroid antibodies (positive thyroid antibodies) in our population sample and mean MBL levels in individuals with positive *versus* negative thyroid antibodies. In the whole group examined (n=120), in patients with hypopituitarism (n=42), in patients with other pituitary diseases (n=57) and in control individuals (n=21), positive thyroid antibodies were found with the following frequencies: 16/85 (18.82%), 4/27 (14.81%), 9/40 (22.50%), and 3/21 (14.29%), respectively (data not presented). As evaluated by the ratio comparison test, the prevalence of positive thyroid antibodies did not differ statistically between the following groups: control vs. hypopituitary patients, *p=*0.923; control vs. other pituitary diseases, *p=*0.454; hypopituitary patients vs. patients with other pituitary diseases, *p=*0.478.

Thereafter, we compared MBL levels between subgroups with positive and negative thyroid antibodies. MBL concentrations did not differ statistically between individuals with positive *versus* negative thyroid antibodies when the statistical comparison was made in the whole group of individuals or in patients with hypopituitarism or in patients with other pituitary diseases or in control individuals; Respective comparisons of MBL levels are as follows: 2231.13 ± 605.94 ng/mL vs. 2134.04 ± 259.46 ng/mL, *p=*0.874; 613.70 ± 223.04 ng/mL vs.1399.36 ± 312.80 ng/mL, *p=*0.315; 1820.67 ± 604.92 ng/mL vs. 2386.34 ± 433.09 ng/mL, *p=*0.520; 5619.05 ng/mL ± 1568.53 ng/mL vs. 2739.16 ng/mL ± 585.68 ng/mL, *p=*0.068 (data not presented).

Summarizing the issue on the possible contribution of positive thyroid antibodies to MBL level it should be stated on the basis of the current results that MBL concentration, in any of groups considered, is not statistically affected by thyroid autoimmunity.

## 4 Discussion

It is well known that patients with hypopituitarism have impaired general health (13, 14). It is highly probable that the immune response might be disturbed, at least or especially *via* secondary adrenal insufficiency (15). That is why it can be hypothesized that certain abnormalities of the lectin pathway of the complement system are already present under conditions of pituitary hormone insufficiency.

Patients with hypopituitarism considered in our study suffered from hypopituitarism of different degree, *i.e.* they had from one to all five pituitary deficiencies. Therefore we discuss shortly particular pituitary deficiencies with relation to our study. Regarding GH deficiency, that one was not confirmed in our patients with the use of insulin tolerance test, being still a gold standard. The reason being is that just till recently we did not have in Poland the reimbursement for the treatment with recombinant human GH (rhGH); however, it has just been confirmed in 2018 (16). It should be recalled that in most cases hypopituitarism develops gradually with GH deficiency being usually the first one, followed by LH/FSH deficiency, TSH deficiency, ACTH deficiency and prolactin deficiency (12, 17). Therefore, it is highly probable that most of our patients with different diagnosed pituitary deficiencies had also GH deficiency (with exception of patients with isolated deficiencies) and that was confirmed indirectly by lower IGF-I levels in patients with hypopituitarism (**Table 1**) and by the observation that lower IGF-I concentrations, similarly to pituitary tropic hormones (*i.e.* TSH, LH and ACTH), were associated with hypopituitarism in the univariate regression analysis (**Table 5**).

The main observation from our study is that MBL level is lower in patients with hypopituitarism comparing to healthy individuals and even to patients with other pituitary diseases (**Figure 2**) and the former relationship was also confirmed by univariate regression analysis (**Table 5**). When the distribution of patients with relation to MBL level was evaluated, no healthy individuals had MBL level below 500 ng/mL whereas almost half of hypopituitary subjects had MBL below this cut-off level.

Such studies have not been performed before. There were only studies concerning relationship between MBL and GH deficiency or excess (9, 10), as it has been already mentioned in the Introduction section. Although IGF-I is the main mediator of GH action on numerous processes, such as linear bone growth and metabolic processes (18), probably it does not mediate GH action on the lectin pathway, because IGF-I treatment did not change MBL level in healthy and GH deficient individuals (9). However, positive correlation between IGF-I and MBL found in our study (**Table 3**) is not in contradiction with the above (9), but simply indicates that low IGF-I level expresses GH deficiency. After all it would be very difficult to confirm the possible correlation between MBL and GH levels due to short half-life of GH making its blood concentration unstable (19).

Not only pituitary tropic hormones, but also peripheral hormones produced by target endocrine glands, contribute to appropriate functioning of the immune system (20–23) and, at the same time, probably also to proper formation of MBL. Such an assumption can be confirmed by results from the present study showing that patients with hypopituitarism being on appropriate replacement therapies (patients with compensated hypopituitarism) had higher MBL levels comparing to those on inadequate replacement therapies (patients with non-compensated hypopituitarism, **Figure 4** and **Table 6**).

Positive correlation found between MBL and glucose concentration in patients with hypopituitarism may be associated with the fact that glucose concentration is positively regulated by two pituitary hormones, *i.e.* GH and ACTH (*via* cortisol). In turn, positive correlation noted between MBL and FT3 in the whole group examined may result from stimulatory effects of thyroid hormones on complement system and MBL level, as it was already mentioned in the Introduction section (6–8). Consistently, the finding that lower FT3 concentrations in patients with hypopituitarism are associated with inadequate replacement therapy, as was documented by univariate regression analysis (**Table 6**), is expected and may result directly from the lack of L-thyroxine therapy in patients with secondary hypopituitarism.

Regarding abnormal lipid profile, the increased total cholesterol was observed more frequently in patients with pituitary diseases, especially in hypopituitary patients, comparing to healthy individuals (**Table 4**), and total cholesterol concentration was found to be the only independent factor associated with hypopituitarism (**Table 5**). Such relationships may result mostly from two pituitary deficiencies, namely GH deficiency (24, 25) and TSH deficiency (causing secondary hypothyroidism) (26). Regarding the latter, such findings are in agreement with what is commonly observed in patients with primary hypothyroidism (26, 27). Even high normal TSH (8, 27, 28) and isolated hypothyroxinemia (29) were associated with abnormal lipid profile in women of childbearing age.

Because a proper supply of vitamin D contributes to appropriate functioning of the immune system (30–32), we checked to what extent vitamin D deficiency changes MBL level. Our patients with hypopituitarism (and also the whole group examined) who had severe vitamin D deficiency were characterized by higher MBL levels than those with recommended vitamin D concentrations (**Table 7**). Therefore such results exclude the possibility that lower MBL level found in our hypopituitary patients may result from vitamin D deficiency. At the same time such results make our observation that low MBL level is directly associated with hypopituitarism much stronger. In turn, rarer occurrence of severe vitamin D deficiency in our hypopituitary patients may possibly result from the assumption that patients who were treated properly with peripheral hormones due to hypopituitarism, did take adequate dose of vitamin D either. It can be mentioned that our observation is in apparent contradiction with results showing that growth hormone deficiency (non-compensated) is associated with vitamin D deficiency (33).

Results obtained in the present study suggest that the degree to which MBL is decreased in patients with hypopituitarism depends on whether the patients were on appropriate replacement therapies and on how many pituitary axes were involved (**Figures 4**, **5** and **Table 2**). Regarding the first point (*i.e.* appropriate vs. inappropriate hormone replacement therapies) it has been statistically confirmed (**Figure 4**) that patients with compensated hypopituitarism have higher mean and median MBL levels when compared to patients with non-compensated hypopituitarism. Because MBL level in compensated patients did not differ statistically from healthy individuals we can state that appropriate hormone replacement therapies improved substantially the functioning of the lectin pathway. In other words, hypopituitary patients on appropriate replacement therapies have the same (or being very close) functioning of the lectin pathway as healthy individuals.

The other mentioned above component which affects MBL level in our hypopituitary patients is the number of pituitary deficiencies. Although no statistically significant differences were found between MBL levels with relation to numbers of pituitary deficiencies, a tendency is seen to the decreased MBL levels with higher numbers of pituitary axes involved. The observation showing that MBL level decreases in patients with four pituitary deficiencies, being especially low in patients with panhypopituitarism, suggests that ACTH deficiency (usually being the fourth deficiency in gradually developing hypopituitarism) resulting in secondary adrenal insufficiency contributes strongly to weakening of the lectin pathway. It should also be mentioned that extremely low MBL level of 96.2 ng/mL was found in our patient with one isolated ACTH deficiency. In this context it can be recalled that the hypothalamic-pituitary-adrenal (HPA) axis is stimulated during the course of certain immune processes (21) and that glucocorticoids, secreted from the adrenal cortex, have pleiotropic (both immunoenhancing and immunosuppressive) effects on the immune system (34, 35), playing also a role in the immune system development (36). In turn, in large prospective study of hypopituitary patients significant increase in the mortality from respiratory infections was reported (13); It is not excluded that the lectin pathway is involved in this last phenomenon because low MBL levels were found to be associated with increased susceptibility to infections (37). Regarding the fifth deficiency, *i.e.* prolactin deficiency, the low efficiency of the lectin pathway (resulting in low MBL level) could be rather associated with severity of the disease such as panhypopituitarism than with low concentration of prolactin by itself. It should be recalled that physiological role of prolactin, except pregnancy and lactation, is not of great significance comparing to other pituitary hormones. However, it is worth mentioning that prolactin participates in the pathogenesis and activity of several autoimmune disorders (38), such as for example systemic lupus erythematosus (39). It should be also mentioned that the single highest values of MBL levels found in our patients with non-compensated hypopituitarism did relate to an individual with three pituitary deficiencies (5891.87 ng/mL) and to another individual with four pituitary deficiencies (4135.07 ng/mL), which is not easy to explain. One should realize that not only low MBL levels but also its higher concentrations may be associated with some pathological conditions as it was confirmed for example in acromegalic patients (9). It can be concluded that due to low number of our patients with particular pituitary deficiencies the current results do constitute the basis for future studies with larger population samples to confirm that high numbers of pituitary deficiencies contribute to lower effectiveness of the lectin pathway.

Because positive thyroid antibodies (similarly to other kind of antibodies) result from the weakening of the immune system and because the association was found between MBL gene polymorphisms and susceptibility to autoimmune thyroid diseases (40) we checked whether the prevalence of positive thyroid antibodies differs between particular groups and whether mean MBL levels differ in individuals with positive *versus* negative thyroid antibodies. The prevalence of positive thyroid antibodies was relatively high, *i.e.* from 14.29% to 22.50%, which can be explained by relatively high age of individuals (41). However, it did not differ between subgroups; It should be especially stressed that very similar percentages of positive thyroid antibodies were found in control and hypopituitary individuals (14.29% vs. 14.81%). Regarding MBL level, no statistically significant differences were found between individuals with positive *versus* negative antibodies independent of the group considered. Both these observations suggest that the thyroid autoimmunity was not the leading causative factor of the decreased MBL level in our patients with hypopituitarism.

Although patients with hypopituitarism were older than control individuals, we did not find any correlation between MBL level and age in any group considered (**Table 2**). Additionally regarding the group of hypopituitary patients and also of patients with other pituitary diseases, mean age of patients with lowest concentrations of MBL (<500 ng/mL) did not differ (was very similar) from mean age of patients with higher levels of MBL (>500 ng/mL) (49.34 ± 4.44 vs. 50.92 ± 3.65 years). Concerning published studies, it is not documented that MBL level is associated with age (42). The only exception is our previous study in which negative correlation was observed between MBL level and age in a specific group of individuals *i.e.* women of reproductive age (8).

The current study has, of course, some limitations. The first one is that the groups considered were not well matched in terms of age, however we confirmed statistically that MBL level was not associated with age in any group considered and, what is of great importance, patients with MBL <500 ng/mL were not older than those with MBL >500 ng/mL. Also in the earlier mentioned study, the group of GH deficient patients and healthy individuals were unmatched (9). The next limitation is that the population sample was relatively small (*i.e.* 120 individuals with 42 patients diagnosed with hypopituitarism). However in studies published so far population samples examined were similarly not very large, comprising 30 healthy individuals, 25 GH-deficient patients, 23 acromegalic patients (9) or four different studies with total group of 108 patients with Turner syndrome and 99 control individuals (10). Another limitation relates to the fact that our patients were not diagnosed with the insulin tolerance test (being a gold standard) to confirm GH deficiency and that they were not treated with rhGH. At last it should be stressed that the only measured parameter of the lectin pathway was MBL concentration so we do not know if decreased MBL level in hypopituitary patients results from decreased formation of this component or its increased consumption. It is also unknown to what extent MBL concentrations are associated with MBL genotypes in particular individuals; as in humans MBL2 genotype is the most important factor affecting circulating MBL concentration (43), it would be valuable to analyze polymorphisms in the MBL2 gene in our patients. Unfortunately, we do not possess appropriate fractions such as the whole blood or frozen clotted blood samples to extract genomic DNA. Therefore at this moment it is impossible to check if MBL concentrations and MBL2 genotypes are distributed in a similar way in the particular groups that are compared, which does constitute a separate limitation.

Taking into account results published so far, MBL level can serve especially as a diagnostic marker (6–8, 44–46). Regarding the application of our current findings, the measurement of MBL level in patients with pituitary hormone deficiencies may be helpful in adjusting optimal doses of appropriate hormone replacement therapies.

According to our knowledge our study is the first one to demonstrate lower MBL level in adults with hypopituitarism of different degree when comparing not only to healthy individuals, but also to hypopituitary patients being on appropriate hormonal replacement therapies. Whether the decreased MBL level in hypopituitary patients reverses alongside replacement treatment remains to be confirmed in longitudinal studies. Additionally, to confirm that high numbers of pituitary deficiencies contribute to lower effectiveness of the lectin pathway larger population samples should be examined. Therefore further studies should be performed to explain direct association between lectin pathway of the complement system with particular pituitary deficiencies and with appropriate hormonal treatment.

Summarizing, hypopituitarism in adults is associated with impaired lectin pathway of the complement system as documented by lower mannan-binding lectin levels. As this phenomenon does not exist in patients with compensated hypopituitarism, it is highly probable that the decreased concentrations of mannan-binding lectin return to normal with the appropriate hormone replacement therapies. Therefore measurement of mannan-binding lectin level in patients with hypopituitarism may be considered as an additional factor helpful to optimize hormone replacement therapies. Additionally, if changes of other components of the lectin pathway are confirmed in hypopituitary patients this will further clarify immune mechanisms of unfavorable consequences of pituitary hormone deficiencies and, consequently, will broaden the knowledge in the field of a relationship between immune and endocrine systems.

## Data availability statement

The raw data supporting the conclusions of this article will be made available by the authors, without undue reservation.

## Ethics statement

The studies involving human participants were reviewed and approved by Ethical Committee of the Polish Mother’s Memorial Hospital - Research Institute, Lodz, Poland (No. 47/2019, 87/2022). The patients/participants provided their written informed consent to participate in this study.

## Author contributions

Conceptualization: MK-L and AEM; methodology: MK-L and AL; software: JS; validation: MK-L; formal analysis: AEM, JS; investigation: AEM; writing—original draft preparation: AEM; writing—review and editing: MK-L; visualization: JS; supervision: MK-L and AL. All authors contributed to the article and approved the submitted version.
